# Can invariant Natural Killer T cells drive B cell fate? a look at the humoral response

**DOI:** 10.3389/fimmu.2025.1505883

**Published:** 2025-02-18

**Authors:** Pablo A. Palacios, Álvaro Santibañez, Fernanda Aguirre-Muñoz, Cristián Gutiérrez-Vera, Valentina Niño de Zepeda-Carrizo, Martín Góngora-Pimentel, Marioly Müller, Mónica Cáceres, Alexis M. Kalergis, Leandro J. Carreño

**Affiliations:** ^1^ Millennium Institute on Immunology and Immunotherapy, Instituto de Ciencias Biomédicas, Facultad de Medicina, Universidad de Chile, Santiago, Chile; ^2^ Departamento de Tecnología Médica, Facultad de Medicina, Universidad de Chile, Santiago, Chile; ^3^ Millennium Institute on Immunology and Immunotherapy, Facultad de Ciencias Biológicas, Pontificia Universidad Católica de Chile, Santiago, Chile

**Keywords:** iNKT cells, glycolipids, B cells, germinal center, class-switch recombination, humoral response, cytokines

## Abstract

Invariant Natural Killer T (NKT) cells represent a unique subset of innate-like T cells that express both NK cell and T cell receptors. These cells are rapidly activated by glycolipid antigens presented via CD1d molecules on antigen-presenting cells (APCs), including B cells, dendritic cells (DCs), and macrophages, or through cytokine-dependent mechanisms. Their ability to produce a wide range of cytokines and express costimulatory molecules underscores their critical role in bridging innate and adaptive immunity. B cells, traditionally recognized for their role in antibody production, also act as potent APCs due to their high expression of CD1d, enabling direct interactions with iNKT cells. This interaction has significant implications for humoral immunity, influencing B cell activation, class-switch recombination (CSR), germinal center formation, and memory B cell differentiation, thus expanding the conventional paradigm of T cell–B cell interactions. While the influence of iNKT cells on B cell biology and humoral responses is well-supported, many aspects of their interaction remain unresolved. Key questions include the roles of different iNKT cell subsets, the diversity of APCs, the spatiotemporal dynamics of these interactions, especially during early activation, and the potential for distinct glycolipid ligands to modulate immune outcomes. Understanding these factors could provide valuable insights into how iNKT cells regulate B cell-mediated immunity and offer opportunities to harness these interactions in immunotherapeutic applications, such as vaccine development. In this review, we examine these unresolved aspects and propose a novel perspective on the regulatory potential of iNKT cells in humoral immunity, emphasizing their promise as a target for innovative vaccine strategies.

## 1 Introduction

Natural Killer T (NKT) cells represent a specialized subset of T cells that integrate features of both the innate and adaptive immune cells. NKT cells are classified into two categories based on TCR diversity: type I NKT or invariant NKT (iNKT) and type II NKT or diverse NKT (dNKT), for the purposes of this article here we will focus on iNKT cells, although dNKT role in immune responses has been addressed elsewhere (1–3). In mice, iNKT cells express a TCR composed of a Vα14-Jα18 α-chain paired with Vβ2, Vβ7, or Vβ8.2 β-chains (4). In humans, iNKT cells possess a TCR consisting of a Vα24-Jα18 α-chain associated with a Vβ11 β-chain (5). This receptor recognizes lipid antigens presented by the non-polymorphic CD1d molecule, rather than peptide antigens presented by major histocompatibility complex (MHC) molecules (6). Upon activation, iNKT cells rapidly produce large quantities of cytokines, including interferon-γ (IFN-γ), interleukin-4 (IL-4), IL-10, and IL-17, enabling them to orchestrate diverse immune responses (7, 8). iNKT cells are capable of activating quickly after antigen encounter, this is in part due to their innate origin, characterized by a pre-activated phenotype, without requiring differentiation or priming by dendritic cells (DCs), unlike conventional T cells (9). Additionally, NKT cells have pre-formed mRNAs, being therefore capable of rapidly produce and secrete several cytokines (10, 11). This phenotype, coupled with their functional heterogeneity, positions iNKT cells as central regulators in various immunological processes, such as anti-tumor activity, pathogen defense, and immune modulation in autoimmune diseases. Recent advances have classified iNKT cells into subsets based on their transcriptional and functional profiles, including iNKT1, iNKT2, and iNKT17, among others, each contributing uniquely to the immune landscape (12, 13).

The relative expression of transcription factor promyelocytic leukemia zinc finger (PLZF) in iNKT cells, has been shown to drive the differentiation into different subsets, and strong TCR signals are shown to regulate PLZF expression (14, 15). In this regard, iNKT1 cells have been classified as PLZF^LOW^/T-bet^+^/RORγt^-^/GATA-3^+/-^, and known to produce IFN-γ, and at low levels IL-4 (16). iNKT1 cells express IL2Rβ, and IL2Rβ-mediated IL-15 signaling is essential for their differentiation (17). iNKT2 have been described as PLZF^HIGH^/T-bet^-^/RORγt^-^/GATA-3^+^, prominently producing IL-4, whereas iNKT17 are classified as PLZF^INT^/T-bet^-^/RORγt^+^/GATA-3^+^, producing mainly IL-17 (18–20). The complete absence of TGF-β signaling led to total loss of RORγt^+^ iNKT17 cells (21, 22). Both iNKT2 and iNKT17 cells express IL-17RB (IL-25 receptor), which is essential for the production of IL-13, IL-9, IL-10, and IL-17 after TCR-mediated stimulation (23), demonstrating that the cytokine production of activated iNKT cells is also influenced by a signal through this receptor.

Studies have also discovered additional subsets, such as iNKT follicular helper (iNKTfh) and iNKT10. Like T follicular helper (Tfh) cells, iNKTfh cells are characterized as Bcl-6^+^/CXCR5^+^/PD-1^+^/ICOS^+^ and produce mainly IL-21 (24, 25); and as to iNKT10, these cells are characterized PLZF^HIGH^/E4BP4^+^, producing IL-10 and therefore having anti-inflammatory role (26, 27). Although the mechanisms of this differentiation remain unknown, the plasticity and heterogeneity of iNKT cells make them a promising target for modulating immune responses in the context of infectious, carcinogenic, autoimmune, and other diseases (12, 13, 28).

A key feature of iNKT cells is their interaction with antigen-presenting cells (APCs), particularly DCs. These interactions not only drive the maturation of DCs through cytokine-mediated mechanisms but also enhance their ability to prime conventional T cells. Through this crosstalk, iNKT cells significantly amplify CD4^+^ and CD8^+^ T cell responses, promoting robust immunity in different contexts. Moreover, the discovery of the glycolipid α-Galactosylceramide (α-GalCer) and its synthetic analogues led to a great understanding iNKT cell biology, allowing researchers to manipulate their activity for therapeutic purposes (29, 30). The activation of iNKT cells by α-GalCer enhances antigen-specific immune responses and has demonstrated its potential in cancer immunotherapy and vaccine adjuvant development, underscoring their clinical relevance.

In addition to their interactions with DCs, iNKT cells play a pivotal role in regulating humoral immunity. iNKT cells influence the activation of B cells through direct engagement via CD1d molecules and promotes their differentiation into germinal center B cells, long-lived plasma cells, and memory B cells, which are essential for sustained antibody production (31–34). This interaction also complements the classical pathway of B cell activation, mediated by Tfh cells. By bridging innate and adaptive immunity, iNKT cells provide critical signals that enhance antibody-mediated responses, with implications in infectious diseases, vaccine efficacy, and autoimmune regulation. This article examines the complex mechanisms by which iNKT cells interact with B cells during the humoral immune response. It highlights how these interactions, along with the involvement of distinct APCs and the use of different α-GalCer analogues, can be harnessed to modulate B cell activation and shape the resulting humoral immune response.

## 2 Functional modulation of iNKT cells by glycolipid ligands

### 2.1 Cytokine bias in the activation of iNKT cells

iNKT cells can produce a diverse array of cytokines, each with distinct roles in modulating immune responses. Multiple mechanisms have been proposed to contribute to the cytokine bias of iNKT cells, including the strength of the interaction with the invariant TCR (35), TCR-dependent stabilization of preformed cytokine mRNAs (11), antigen presentation by distinct APCs (36), location where the antigens are loaded onto CD1d molecules and whether they are presented in lipid rafts or not (37). In this regard, plasma membrane glycolipid rafts facilitate α-GalCer presentation on CD1d, which are also required for efficient signal transduction, specially at low ligand densities (38, 39). In addition, microenvironmental signals may also influence iNKT cell activation considering their tissue-specific distribution (19, 40, 41).

Irrespective of this debate, the existence of different iNKT cell subsets is clear and as mentioned initially, they have a signature expression of transcription factors and cytokines, which may be targeted by different glycolipid ligands.

α-GalCer is the most studied glycolipid ligand of iNKT cells due to its remarkable activating properties, which have led to significant advancements in studying this non-convential population of lymphocytes (42). Although initial studies showed that this glycolipid had promising results in promoting anti-tumoral activity and pathogen-specific immunity, the simultaneous production of cytokines with opposite properties such as IL-4 and IFN-γ (43), represents a counterproductive effect considering that this may elicit unpredictable immune responses (44–46).

Modifying the backbone of α-GalCer can result in significant changes in cytokine profiles produced by iNKT cells. The development of synthetic α-GalCer analogues, either through experimental or in silic designs (47) that can induce a Th1- or Th2- biased cytokine response holds therapeutic potential for treating conditions such as pathogen infections, autoimmunity, cancer, and allergies, where imbalanced or polarized cytokine production often drives disease pathogenesis (42, 48, 49).

### 2.2 α-GalCer analogues modulate iNKT cell activation

Among the synthetic ligands known to polarize toward a Th2-like response or that induce iNKT2 cell activation, OCH is one of the most studied. This glycolipid contains a truncated acyl and sphingosine chains, inducing a higher production of IL-4 upon injection in mice when compared to α-GalCer, with mild or non-detectable production of IFN-γ, which is observed in both mouse and human iNKT cells, being used effectively in autoimmune murine models (13, 50–53). Recently, OCH has been used in SARS-CoV-2 studies showing that prevents has therapeutic effects in the late stage of infection (54), and also, the first-in-human clinical trial of this glycolipid in the context of multiple sclerosis showing promising results (55). On the other hand, the analogue α-GalCer C20:2 has a truncated and unsaturated acyl chain, inducing higher production of IL-4 in comparison to α-GalCer, and although it does induce the production of IFN-γ, levels are lower compared to α-GalCer, however, these results have been observed only in mouse iNKT cells (56–58).

Regarding analogues proven to induce a Th1-like response or promoting iNKT1 cell activation, most studied are α-C-GalCer and 7DW8-5, and to a lesser extent the recently reported glycolipids AH10-7 and C34. α-C-GalCer has a CH_2_-based glycosidic linkage rather than the oxygen-based glycosidic linkage of α-GalCer, which promotes a higher production of IFN-γ and IL-12, with almost non-detectable production of IL-4 compared to α-GalCer, although these effects has been observed only in mouse iNKT cells (49, 59, 60). Additionally, this glycolipid was shown to be up to 1000-fold more potent than α-GalCer in terms of causing protection against malaria, influenza virus, and melanoma metastasis in mouse models (49, 59, 61), and also being used in the design of BCG vaccine (62). Further studies by Tsuji’s group led to the identification of several C-Glycoside analogues, in which the galactose had an a-linked E-alkene connecting to the ceramide portion, inducing strong IFN-γ production in both mouse and human iNKT cells (63). 7DW8-5 has a fluorinated benzene ring at the end of a C8 length fatty acyl chain, which generates a higher activation of both mouse and human iNKT, with higher IFN-γ production, when compared to α-GalCer, being used in cancer studies (64–66). This glycolipid has demonstrated a superior adjuvant effect compared to α-GalCer in HIV and malaria vaccines in mice, and recently it has been shown to block SARS-CoV-2, respiratory syncytial and influenza virus in mice and hamsters (64, 67).

The glycolipid AH10-7 has also shown promising results. This glycolipid has a modification in the galactose, with a hydrocinamoyl ester group on carbon 6, and also lacks the hydroxyl group on carbon 4 of the sphingosine, leading to an overall response polarized toward IFN-γ production by mouse and human iNKT cells, and showing strong anti-tumoral effect against B16-F10 melanoma (68). This glycolipid has also proven to be effective in a partially humanized mice model expressing human CD1d (68). As to C34 analogue, it contains two phenyl rings on the acyl chain compared to α-GalCer and elicited a strong IFN-γ production, with anti-tumoral effects against breast, lung, melanoma, and neuroblastoma cancer (69, 70). Recently, several analogues have been designed using computational analysis and a humanized mouse model in which cells express the human αTCR chain sequence and human CD1d, aiming to improve the identification of strong iNKT cell agonists for subsequent clinical trials (71).

More recently, diether moieties have demonstrated a structure-activity relationship that selectively promotes the secretion of IL-17 over other cytokines. This finding suggests potential protective effects against pathogens, likely driven by iNKT17 cells (72). Numerous intriguing analogues have been developed to polarize the iNKT cell response (73), although the specific mechanisms by which they are processed and presented onto CD1d molecules remain unclear for most of them (37, 74–76).

Although some reports have shown that structural analogues of α-GalCer enhance the humoral response, it has not yet been discussed whether differential activation of iNKT cells by different glycolipids ligands can modulate the outcome of B cell activation and ultimately antibody production.

## 3 Where and how do iNKT and B cells interact?

In mice, iNKT cells become detectable in the thymus by days 5–6 after birth and in peripheral tissues starting around day 8 (6, 77). Later in adulthood, the frequency of iNKT cell from total lymphocytes is 12-30% in the liver, 1-3% in the spleen, 5-10% in the lungs, 0.5-1% in the thymus, 0.4-8% in the bone marrow, 0.2-1% in lymph nodes, 0.05-0.6% in the intestine and 0.2% in the blood (78–82). As has been suggested previously, their location defines their features and functions (41).

Peripheral iNKT cells exhibit tissue-specific characteristics and interactions. In adipose tissue, they predominantly engage with CD1d-expressing adipocytes, macrophages, and DCs, with additional interactions involving eosinophils, regulating pro and anti-inflammatory signals in obesity-associated inflammation (83–85). In the lungs, iNKT cells interact with various APCs, including alveolar macrophages, CD11b^+^ DCs, CD103^+^ DCs, and monocyte-derived DCs, playing a crucial role in host defense against pathogens and allergic asthma (28, 86, 87). In the intestine, they primarily engage with CD1d-expressing CD11c^+^ cells, which play a critical role in maintaining spatial separation between the microbiota and epithelial cells (88). In the liver, iNKT cells are predominantly localized on the luminal surface of sinusoidal endothelial cells, where they interact with CD1d-expressing Kupffer cells (89). Beyond these tissue-specific roles, iNKT cells contribute significantly to immune functions within lymphoid tissues such as the spleen and lymph nodes.

### 3.1 Dynamics between iNKT cells and CD1d-expressing APCs in secondary lymphoid organs

The spleen is a highly organized organ consisting of red and white pulp, with the latter serving as the primary residence for mature T and B cells, localized within distinct regions known as the T and B cell zones, respectively. This organ acts as a blood filter, maintaining continuous contact with blood-borne antigens (90). Between the red and white pulp exists an area called marginal zone (MZ), which lies just outside of the lymphocyte-residing white pulp and contains MZ B cells, DCs, MZ macrophages (CD209b^+^, MARCO^+^, SR-A^+^, ER-TR9^+^) and metallophilic macrophages (Siglec-1^+^, CD68^+^) (90–95). These APCs are the main ones responsible for initiating an immune response against particles and pathogens in the blood (95–97). Humans also have a structure similar to mice MZ which is defined as perifollicular zone, surrounding B cell zones where MZ B cells are located, and therefore their characteristics differ (98).

During resting state, splenic iNKT cells are widely distributed throughout the parenchyma, including the T and B cell zones, and around the MZ (24, 99). These cells exhibit unique recirculation and homing properties between B and T cell zones, driven by a combination of molecular mechanisms. The transcription factor PLZF promotes the expression of integrins such as LFA-1, which, in conjunction with its ligand ICAM-1, facilitates the residence of iNKT cells in extravascular areas and the T cell zones of the spleen and lymph nodes (100). This localization is further refined by chemokine signaling and other microenvironmental factors (41). In response to exogenous stimulation, such as α-GalCer or *Sphingomonadaceae*-derived glycosphingolipid (GSL-1) administered intravenously, iNKT cells are recruited to the marginal zone within 4 hours, and around 8 hours when mice are infected with *S. pneumoniae*, however, when addressing indirect or cytokine-mediated activation through the administration of IL-12 and IL-18, these cells distribute homogenously in the different zones (99). Disruption of MZ severely impacts iNKT cell activation (101), so altogether this indicates that during early activation, iNKT cells migrate to MZ requiring antigen presentation by CD1d-expressing APCs facilitating their rapid access to blood-borne antigens.

**Figure 1:**
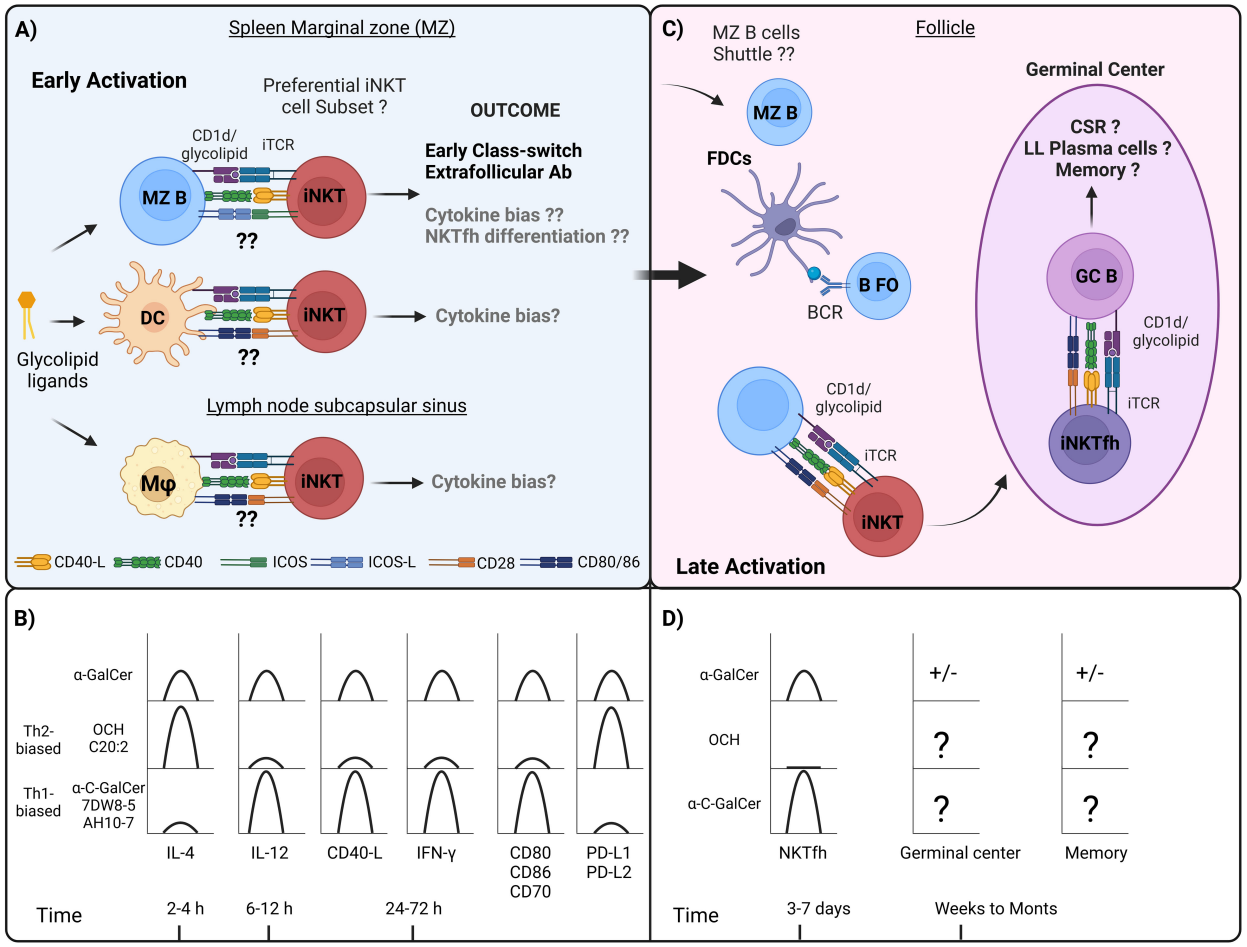
Interactions between APCs and iNKT cells during the course of humoral response, and differences in cytokine response, costimulatory molecules, and iNKTfh formation by different α-GalCer analogues. **(A)** In the first hours and days following the administration of the iNKT cell ligand, it has been observed that at the level of secondary lymphoid organs, iNKT cells migrate to the spleen marginal zone (MZ) and concentrate in this area, which also occurs in the subcapsular sinus zone of the lymph nodes. Diverse APCs that express CD1d are present in these areas, including MZ B, DCs, MZ macrophages, and metallophilic macrophages. Interactions between MZ B and iNKT cells have been shown to depend on ICOS and ICOS-L signaling, which primarily promotes IL-4 production. In addition, early MZ B cell activation would generate an extrafollicular response and early class-switch recombination. Regarding DCs, and specifically CD8^+^ subset, this interaction has been shown to be dependent on CD40-CD40-L, CD28-CD80/CD86, which generally induces the production of both IL-4 and predominantly IFN-γ. As to MZ macrophages, there appears to be a predominant induction of IFN-γ, whereas subcapsular sinus macrophages, it is not clear yet, however, in the context of viral response are associated with iNKT-mediated IL-4 production. **(B)** While it remains unclear whether different APCs differentially process α-GalCer analogues or direct antigen presentation toward specific iNKT cell subsets, these analogues have been reported to influence the kinetics of cytokine production and the expression of costimulatory molecules. Th2-biased α-GalCer analogues such as OCH and C20:2 predominantly stimulate the production of IL-4 in mouse iNKT cells, however, in the case of human iNKT cells, only OCH has been shown to induce this effect. In the case of OCH it has been shown to promote the expression of PD-L1 and PD-L2 in DCs. Th1-biased α-GalCer analogues, such as 7DW8-5 and AH10-7 predominantly stimulate the production of IFN-γ and IL-12 in mouse and human iNKT cells, and only in mouse iNKT cells in the case of α-C-GalCer, and although not evaluated it is proposed that these analogues promote the expression of CD40-L. It has been reported that α-C-GalCer promotes an increase in the expression of CD80, CD86 and CD70 in DCs. **(C)** In the later stages, between 3 and 7 days after iNKT cell activation, iNKTfh cells are induced through interactions with B cells. Depending on whether this interaction is cognate or non-cognate, it can drive CSR, germinal center formation with further CSR, and the generation of long-lived (LL) plasma cells along with memory B cells. **(D)** The induction of iNKTfh cells has been addressed after stimulation with different glycolipids, and only α-GalCer and α-C-GalCer were shown to induce the generation of these cells, being higher for α-C-GalCer, whereas OCH wasn´t capable to induce this phenotype. ± It is controversial whether α-GalCer stimulation promotes germinal center and memory response in the absence of Tfh cells. As to other Th1- or Th2-biased glycolipid analogues indicated in the figure, it has not been addressed whether they induce the formation of germinal center and memory B cell responses.

Key questions in this dynamic are which specific MZ-resident APCs interact with iNKT cells during the initial activation phase and how these interactions influence iNKT cell activation shaping the resulting humoral response (**Figure 1**).

Among professional APCs, MZ B cells are those that express the highest levels of CD1d, and also high levels of costimulatory molecules such as CD40, CD80, B7-H1 and ICOS-L (102). Initially, *in vitro* assays using spleen sorted MZ B cells for α-GalCer antigen presentation showed that these cells required DCs to promote the activation of lin^–^CD4^+^CD3^+^NK1.1^int^ sorted NKT, having a collaborative role in this process (103). However, latter assays have pointed out that MZ B cells induced higher proliferation of NKT cells when compared to conventional DCs (cDCs), Follicular B cells (FO B) and B-1 B cells (32, 102). Additionally, MZ B cell-mediated activation of NKT cells leads to a significantly higher production of IL-4 during the first 4 hours compared to cDCs, with IL-4 levels becoming comparable at 16 and 72 hours. IFN-γ production exhibits the opposite pattern, since only cDC-mediated activation promoting its production at 16 and 72 hours, whereas both APC promoted the production of IL-13 (102). Interestingly, ICOS-ICOSL blockade in these assays selectively inhibited IL-4 and IL-13 production (102). This has also been reported previously in ICOS^-/-^ iNKT cells, however, it also affected the production of IFN-γ, IL-10 and IL-5 (104).

As to macrophages and DCs, experiments using spleen-enriched SIGN-R1^+^ macrophages and CD11c^hi^ DCs loaded with α-GalCer, efficiently activate NKT cell hybridoma DN32.D3. In line with these results, mice treated with clodronate liposomes (CLL), which depletes MZ macrophages, metallophilic macrophages, and DCs from the MZ and red pulp, there is a significant reduction of IFN-γ-producing iNKT cells (101). Same results were shown in other study, however, three weeks after treatment with clodronate, when DCs had been restored in the MZ, IFN-γ^+^ iNKT cells were recovered (~ 40%), but no the maximum level observed with no depletion (~ 50%), showing that although DCs are important for IFN-γ production by iNKT cells, macrophages could act synergically with these cells (99).

As to the contributing of these cells to IL-4 production by iNKT cells, further studies using mixed bone marrow chimeras—where B cells lacked CD1d expression—showed no alteration in IL-4 production by splenocytes in response to *in vivo* administration of α-GalCer. Additionally, α-GalCer administration 24 hours after treatment with clodronate resulted a complete reduction of IL-4 production by splenocytes (from 0.5% to <0.1% approximately) (99). However, when examining IL-4 production specifically by iNKT cells (CD1d-tetramer^+^ splenocytes), the same experiment revealed that administering α-GalCer 24 hours after clodronate treatment significantly reduced IL-4 production by iNKT cells (from 20% to 5% approximately). Despite this, a small percentage of IL-4^+^ iNKT cells remained (5% compared to 0-1% observed in the treatment with vehicle). In contrast, three weeks post-treatment with clodronate, when DCs had been restored in the MZ, IL-4 production was recovered almost to maximum level observed with no depletion (99). These findings highlight the critical role of DCs in IL-4 production by iNKT cells and suggest that MZ B cells could also contribute to this process.

The role of CD8^+^ DCs has been shown to be essential for early iNKT cell activation. These cells are critical for iNKT mediated IFN-γ production in response to pneumococcal infection (99). This has also been addressed by another study by using L363 mAb, which recognizes CD1d/α-GalCer complex, where CD8^+^ DEC205^+^ DCs were shown to be the main population taking up exogenous glycolipid antigens when administered intraperitoneally and mainly CD8^+^ and CD8^-^ DCs promoted the expression of CD69 in iNKT cells, whereas B cells didn’t (105). *Batf3^-/-^* mice lacking CD8^+^ DCs, generated less serum levels of IFN-γ and IL-4 when compared to WT mice, however, there wasn´t a complete reduction in the production of these cytokines, suggesting the presence of compensatory mechanisms by other APCs (105).

Lymph nodes (LNs) are other important secondary lymphoid organs and also organized like filters to capture antigens. They house various lymphoid and myeloid cells that transport particulate material from the afferent lymph into the subcapsular sinus of the lymph node (106). In popliteal LNs at steady state, endogenous iNKT cells localize in the interfollicular region and medulla but not in the T cell-rich paracortex (51, 107), and when they are activated with silica particles coated with antigenic lipids, they migrate to make contact with CD1d-expressing CD169^+^ macrophages lining the subcapsular sinus (91). This is critical during the initiation of antiviral B cell mediated immunity, since macrophages prime iNKT cells in the interfollicular areas promoting an early production of IL-4 necessary for appropriate immune response (108).

The redistribution of activated iNKT cells leads to the contact-dependent maturation of macrophages, which can limit potential pathogen spreading in secondary lymphoid organs, and of DCs, which relocate to T cell zones and promote downstream adaptive T and B cell responses, resulting in the so-called non-cognate iNKT cell help, as will be addressed in the next sections (50, 52).

### 3.2 Role of costimulatory pathways in the activation of iNKT cells

Regarding classical costimulatory pathways involved in iNKT cell activation such as CD28-CD80/CD86 and CD40-CD40L (CD154), CD28^-/-^ mice receiving an intraperitoneal administration of α-GalCer showed reduced production of IFN-γ and almost no production of IL-4 compared to WT mice. On the other hand, CD40^-/-^ mice showed reduced production of IFN-γ, and notably, and enhancement on IL-4 production compared to WT mice (109). *In vivo* assays have shown that Th2-biased analogue OCH generates a lower expression of CD40L compared to α-GalCer, although the kinetics of expression it’s not clear, since some reports showed an early expression at 2 hours whereas others show a peak induction at 24 hours (56, 110, 111). Further assays showed that the absence of CD40-CD40L and IFN-γ signaling in the treatment with OCH results in no systemic production of IL-12. Additionally, simultaneous administration of OCH and IL-12 promotes IFN-γ production in iNKT and NK cells (110). This suggests that Th1-biased analogues might promote the expression of CD40-L in iNKT cells.

Other reports have evaluated the expression of costimulatory and coinhibitory molecules on CD11c^+^ CD8^+^ DCs *in vivo* after intraperitoneal administration of α-GalCer, OCH and α-C-GalCer. Both α-GalCer and α-C-GalCer promoted an increase in the expression of CD70, CD80, CD86 and Rae-1 at 20-40 hours, being higher for α-C-GalCer, whereas OCH only induced a slight increase in CD80 and Rae-1 (105). On the other hand, OCH promoted an increase in the expression of PD-L1 and PD-L2 20 hours after immunization, whereas α-GalCer and α-C-GalCer only generated a mild increase in the expression of PD-L1, being lower for α-C-GalCer (105). These results support the regulatory role of costimulatory molecules in APCs controlling the outcome of iNKT cell activation during early stages, although it is still unclear whether this applies to other APCs and if this result from engaging different iNKT cell subsets.

Together these findings suggest that during early stages, distinct APCs may engage iNKT cells and with specific costimulatory molecules, with each specific APC influencing the activation of different iNKT cell subsets, based on the observed cytokine profiles. For instance, MZ B cells predominantly mediate the production of IL-4 and IL-13, which may be driven by iNKT2 activation by engaging ICOS-ICOSL and PD-1-PD-L1/PD-L2. At the same time, cDCs promote both the production of IFN-γ and IL-4, indicative of iNKT1 and probably iNKT2 activation, regulated through CD28-CD80/CD86, CD27-CD70 and NKG2D-Rae-1 signaling. Still, it is most likely that multiple APCs can synergistically enhance iNKT cell responses, collectively shaping their immunological outcomes (**Figures 1A, B**).

Despite this proposed model, the precise relationships between APCs, cytokine outputs, and the full spectrum of activation of iNKT subsets by a thoroughly characterization using signature markers for each one have not been addressed yet to be fully characterized. Importantly, initial interactions between iNKT and APCs might have an impact on early humoral response, probably through MZ B cells.

### 3.3 Follicular and marginal zone B cell responses

Secondary lymphoid organs predominantly contain two subsets of B cells: FO B cells, and as mentioned previously, MZ B cells. FO B cell are present in circulating B cells in the bone marrow and blood (112). Although these cells are present in mice and humans, their surface markers exhibit different expression patterns (113, 114). These cells are the most abundant and are located mainly in the follicles of secondary lymphoid organs. These cells are responsible for primarily responding against protein antigens with the assistance of Th cells; therefore, this response is classified as T-dependent, where they have been shown to contribute to the formation of germinal center, class-switch recombination (CSR), and somatic hypermutation (SHM), leading to affinity maturation and the production of high-affinity antibodies within days to weeks (115, 116).

T cell-activated B cells will seed the germinal center located in the center of the follicle, where they will initiate rapid proliferation. At the same time, two compartments, known as Light Zone (LZ) and Dark Zone (DZ), are being developed (117). In the DZ, B cells in the fast division, known as centroblasts, undergo SHM of the genes encoding their BCR (118, 119). Once germinal center B cells have undergone SHM in the DZ, they will migrate to LZ to receive positive selection signals from Tfh cells and FDCs (120, 121) because this mutational process can be deleterious to the centroblasts. The selection signal will ensure that only B cells bearing a BCR with an improved affinity for antigen differentiate into long-lived antibody-secreting cells and memory B cells (116).

Regarding CSR, this is an intrachromosomal DNA rearrangement of the immunoglobulin IgM-IgD heavy-chain locus in B cells when activated either in the extrafollicular zone or within germinal centers (122). As a result of this process, B cells will be able to express different isotypes of antibodies including IgG subtypes, IgA, or IgE, without altering their specificity for the antigen (123).

MZ B cells also play a crucial role in humoral response as they are strategically positioned to serve as the first line of defense against blood-borne pathogens and systemic antigens (94). In humans, these cells circulate, whereas in mice, they reside in the spleen’s marginal zone, a crucial area between the bloodstream and lymphoid tissue (124). MZ B cells are distinguished by high CD21/35 receptor expression, which corresponds to complement receptor 2, and low expression of the CD23 receptor (CD21^HIGH^CD23^LOW^). Notably, the CD21/35 receptor forms a complex with the B cell receptor (BCR) and CD19 receptor, reducing the activation threshold of these cells. This lowered activation threshold enables MZ B cells to respond rapidly upon antigen recognition, providing a distinct advantage over other B cell subsets (125).

This subset can produce rapid and early immune responses in minutes to hours once they encounter microbes or exogen particles. Their phenotype is heterogeneous, composed of naive and memory B cells, and are particularly important in T-independent humoral response, consisting mainly of non-protein polymeric antigens, and their recognition will promote their rapid differentiation into short-lived extrafollicular plasma cells to generate low-affinity antigen-specific IgM or in some cases IgG3 (94, 126). Memory B cells, also located in the MZ, participate in recall T-dependent responses after immunization with the same antigens (127).

Interestingly, MZ B cells can differentiate into FO B cells in response to T-dependent antigens, shuttling between the marginal zone and follicles to deliver blood-borne antigens to follicular DCs (FDCs) (128, 129).

As addressed in the previous section, during the early stages of a productive humoral immune response, iNKT cells are likely interacting with MZ B cells, either through cell contact-dependent or independent, modulating extrafollicular antibody production. As will be addressed next, iNKT cells require B cells to differentiate into iNKTfh cells and thus be able to migrate to the follicles and interact with FO B cells, influencing their activation and the subsequent germinal center responses (**Figure 1C**). Interestingly, various α-GalCer analogs have been found to elicit diverse effects on the differentiation of iNKT cells into iNKTfh cells, which may impact on the quality and outcome of the humoral response (**Figure 1D**).

Considering that iNKT cells are also located in the peritoneum where B-1 B cells reside, we wanted to address, although briefly, the importance of this subset and the impact of iNKT cells in their function. Similar to MZ B cells, B-1 cells are associated with T-independent humoral response, since their capacity to produce natural antibodies to the blood stream and to respond rapidly after antigen encounter (130). Although these cells are also present in the spleen, their main location is pleural and peritoneal cavities in mice (90, 131). An interesting connection between iNKT cells and B-1 cells has been identified in the context of cutaneous contact sensitivity, where this interaction plays a key role in the initiation of this response (132–134). Contact sensitivity activates iNKT cells, prompting them to produce IL-4, which in turn coactivates B-1 cells, leading to the production of antigen-specific antibodies. This mechanism is particularly significant in allergic and autoimmune diseases, where infections can exacerbate T cell responses to allergens or autoantigens, potentially worsening disease symptoms (132).

The majority of CD5^+^ B cells located in the peritoneal cavity are known to express CD1d and are closely linked to autoimmune diseases (135). In this line, CD1d-expressing B-1 B cells are reported to produce IL-10, having a regulatory phenotype, commonly linked to regulatory B cells (Bregs) (136–138). Interestingly, CD1d knock mouse, have reduced frequency of IL-10-producing B cells in the spleen and peritoneal cavities, compared to wild type mouse, which also produce higher levels of proinflammatory cytokines (139). In contrast, studies on the pathological accumulation of CD5^+^ B cells, such as in chronic lymphocytic leukemia (CLL), have shown that CD1d expression and iNKT cells are not essential for the development, expansion, or IL-10 competence of CD5^+^ B cells in mice prone to benign or leukemic CLL-like B cell proliferation (140). However, studies examining the impact of iNKT cell absence on the formation and function of B-1 cells in non-pathological contexts, such as in Traj knock-out mice (which lack iNKT cells), have not yet been conducted.

### 3.4 Cognate and non-cognate help between iNKT and B cells

iNKT cells promote productive humoral responses by assisting B cells through two well-characterized mechanisms: cognate and non-cognate interactions.

Cognate help involves direct cell-to-cell interaction between iNKT and B cells. This process has been evaluated through BCR-mediated antigen engagement to promote lipid internalization and subsequent presentation by the CD1d molecule, facilitating the interaction between both cells (34). These interactions promote the formation of extrafollicular plasma cell foci, enhancing antibody responses, and also limited germinal center formation in the absence of Th cells (32, 34, 141).

Cognate interactions also drive the differentiation of iNKT cells into iNKTfh cells. These cells closely resemble Tfh cells, expressing transcription factor Bcl-6, which regulates their migration via chemokine receptors (24, 142). Both human and murine iNKTfh cells express markers such as CD4, CXCR5, PD1, and IL-21 (143, 144). Within follicles, these cells engage in prolonged interactions with B cells, mediated by SLAM-SLAM interactions in a signaling lymphocyte activation molecule (SAP)-dependent manner, along with costimulatory signals from CD40L and CD28 (31, 32, 114, 145).

**Figure 2:**
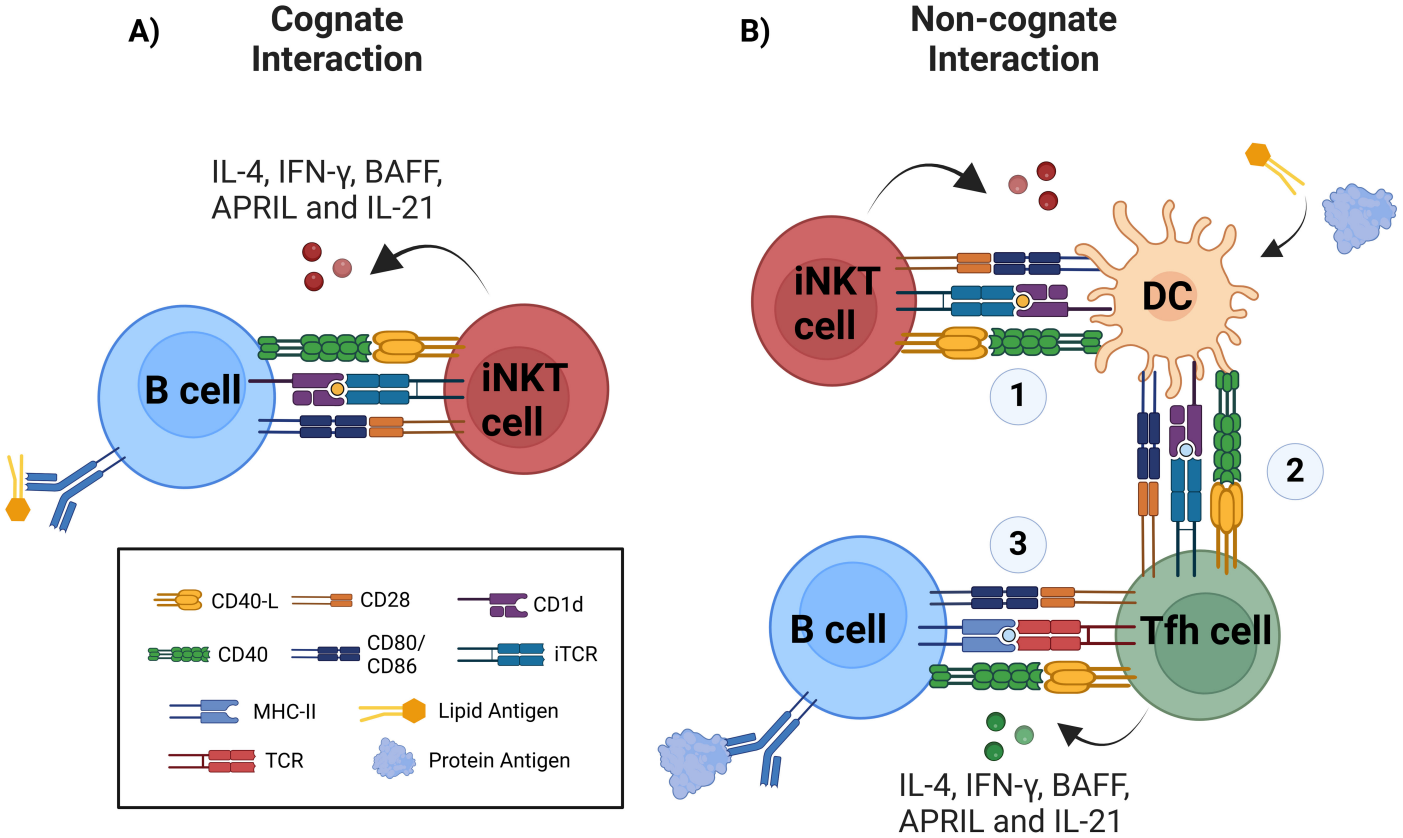
Improvement of humoral responses via iNKT cell activation occurs via cognate and non-cognate interactions. **(A)** During cognate help, B cells uptake and present glycolipid antigen in CD1d molecule to iNKT cells, this direct interaction via CD1d/glycolipid complex and TCR is supported by costimulatory molecules such as CD40/CD40-L, CD28/CD80-86, and other costimulatory signals, triggering iNKT cell activation and cytokine production including IL-4, IFN-γ, APRIL, BAFF, and IL-21. B cell activation results in extrafollicular plasmablast, early class-switch recombination (CSR), early germinal-center formation, and regarding memory response, there is controversial data. **(B)** Non-cognate or indirect help is triggered against protein antigens when using glycolipids as adjuvants, therefore requiring initial activation of iNKT cells by CD1d-expressing glycolipid-presenting DCs (1) and further DCs licensing to promote antigen presentation to CD4^+^ T cells via MHC-II. (2) Antigen-specific Th cell activation and differentiation into Tfh cells. (3) Finally, canonical activation of B cells is initiated. Protein-specific B cells will receive help from Tfh cells, resulting in the generation of plasmablasts, germinal centers, robust affinity maturation, class-switched antibody production by plasma cells (PC) and memory B cells.

The formation of iNKTfh cells requires Bcl-6, CD28, CD1d, and B cells, since in their absence the generation of this phenotype and cognate interaction is abrogated (24, 25). In addition, cognate help requires IL-21 production by iNKT cells, otherwise antibody production is reduced (144). Cytokines such as IFN-γ, IL-4, BAFF and APRIL are also produced by iNKT cells during cognate interaction and are especially important for CSR and long-term plasma cell survival (**Figure 2A**) (32, 146, 147).

While this mechanism accelerates the primary IgG response through germinal center-like structures and affinity maturation, it typically does not produce long-lived plasma cells or memory B cells, with some exceptions (148, 149). In these cases, the antigen was delivered through liposomal nanoparticles containing the NKT cell ligand and coated with antigens on their surface, suggesting that particulate delivery of the glycolipid antigen would promote these processes.

Non-cognate help occurs when iNKT cells indirectly promote B cell responses. This is often observed during immune responses to protein antigens in the presence of iNKT ligands, such as α-GalCer, used as adjuvants. In this mechanism, DCs presenting glycolipid antigens on CD1d interact with iNKT cells, leading to DC “licensing.” Licensed DCs upregulate MHC-II, CD40, and other costimulatory molecules, enabling them to activate naïve CD4^+^ T cells. These T cells subsequently differentiate into Tfh cells, which interact with B cells to drive CSR, germinal center formation, long-term antibody production, and memory B cell generation (**Figure 2B**) (150, 151).

Other APCs, such as CD169+ macrophages, can also be activated via CD1d-antigen interactions and IL-18 secretion. This activation facilitates iNKT cell migration to follicular borders, where they release IL-4 to support early germinal center formation (91, 108). However, these interactions do not induce an iNKTfh phenotype (152).

Both cognate and non-cognate interactions involve cytokine-mediated processes that drive DC licensing and CSR in B cells (52). Licensed DCs activate naïve T cells into Tfh cells, allowing both iNKTfh and Tfh cells to cooperatively activate B cells (153). iNKT-mediated responses can alter T-independent B cell activation. For example, glycolipid-containing antigens internalized via BCRs or low-density lipoprotein receptors (LDL-Rs) are presented on CD1d molecules by B cells, facilitating cognate interactions with iNKT cells (32, 34, 154). This bypasses the typical T-independent response, which usually produces short-lived IgM antibodies without CSR or germinal center formation. iNKT ligands can induce iNKTfh cells in a T-independent context, promoting processes similar to T-dependent responses, such as enhanced antibody production, germinal center-like activity, and affinity maturation. These mechanisms result in high titers of specific IgM and class-switched antibodies, albeit short-lived and without memory formation (24, 99, 141).

By integrating both direct and indirect pathways, iNKT cells significantly enhance B cell responses, highlighting their versatile role in shaping humoral immunity.

## 4 Can the differential activation of iNKT cells influence the B cell fate?

### 4.1 T helper functions in germinal center and CSR

B cells that have received T cell help may undergo a variety of differentiation states as short- and long-live plasma cells with high-affinity antibodies and memory B cells. Interaction between both cells is critical to induce germinal center formation into secondary lymphoid organs upon and invader pathogens or after the immunization with a T-dependent antigens. To initiate germinal center development, B cells must first recognize the antigen directly via their BCR or on the surface of FDCs (155, 156). Activated B cells will migrate to the interface between the B cell follicle and the T cell zone. There, B cells are ready to present the peptides derived from the antigens, and endocytosed previously, on MHC-II molecules to Th cells, which provide them with costimulatory survival signals (157, 158).

The first cognate interaction between Th cells and naive B cells occurs when lymphoblasts are generated before germinal center formation, which is when CSR initiates (122, 159).

The activation of extrafollicular B cells produces an early neutralizing antibody response mounted by short-lived plasma cells, which is necessary to control the spreading of an infection on time (108). Although T-independent stimuli, like polysaccharides or TLR agonists, can participate in short-lived plasma cell formation (126), cognate interactions with antigen-specific Th cells greatly facilitate CSR (160). Notably, only class-switched plasmablasts derived from germinal center selection can give rise to long-lived plasma cells, which either migrate to the bone marrow to receive survival signals or remain in the follicles where they originated (161).

A critical component of CSR is the enzyme Activation-Induced Cytidine Deaminase (AID), known as Aicda in mice or AID in humans, which is responsible for rearrangements in the IgH locus of B cells. The Ig heavy chain IgM-IgD locus has distinct promoters containing elements responsive to various transcription factors, primarily induced by BCR, CD40, and cytokines. These elements lead to the transcription of germline transcripts (GLTs), essential for CSR, and determine the antibody isotype that will be produced (122, 162). Cognate interactions with Th cells provide two types of CSR-inducing stimuli. The primary CSR stimuli are mediated by CD40L, which increases its expression following T-cell activation and induces AID transcription (114). Cytokines command the secondary CSR stimuli and will drive this process toward the best isotype required. Two of the significant cytokines secreted by Tfh cells are IL-4 and IL-21. Both cytokines are crucial for selecting high-affinity antibody-producing B cells and expressing central genes in CSR, such as Bcl-6 and Aicda (163, 164). Cytokines such as IFN-γ and IL-10 can also be produced by Tfh cells (165, 166). In the case of IL-4 and IFN-γ, they have been described to promote CSR toward IgG1 and IgG2a/c isotypes in mice, respectively, highlighting their close cooperation (167, 168).

### 4.2 Improvement of T helper responses by iNKT cells

Tfh cells are uniquely equipped to support germinal center reactions; however, their differentiation occurs through multiple stages (169). The first step begins when Th cells are primed by costimulatory signals and peptides loaded in MHC molecules of resident DCs, known as licensed DCs, and then they migrate towards the border between T and B cells. In the second step, migratory cDC2 (CD11b^+^ CD8α^-^) cells that reside in this site will support pre-Tfh cell differentiation alongside other migratory DCs through the expression of ICOSL and OX40L (170, 171). Finally, In the third step, activated Th cells upregulate the transcription factor Bcl-6 (172) and the chemokine CXCR5 to migrate toward the border of follicles where SAP-dependent interactions with activated B cells being the major APC in this final step to complete Tfh cell differentiation (142).

The α-GalCer-activated iNKT cells contribute to DC licensing *in vivo*, resulting in increased cell surface expression of MHC-II, the costimulatory molecules CD40, CD80, CD86, and the endocytic receptor DEC-205 (150). As mentioned previously, CD8α^+^DEC-205^+^ DCs are the most competent presenters of glycolipid antigens *in vivo*, and for a range of α-GalCer analogues that polarize the cytokine responses. Th1- or Th2-biased glycolipids led to markedly different changes in the expression of costimulatory and coinhibitory molecules on these cells in response to various chemical forms of α-GalCer, as addressed in the previous sections (105). The interaction between iNKT cells and DCs is bidirectional through direct interaction and cytokine production (173). Thus, direct cellular contact between DCs and iNKT cells in a CD40-CD40-L-dependent manner provides a strong feed-forward signal depending on the chemical structure of the CD1d ligand as well as the nature of the APC (150).

Moreover, the interaction of NKT cells with immature DCs promotes tolerance, while mature DCs promote IFN-γ and IL-4 by NKT cells (174). iNKT cells constitutively express the IL-12 receptor, and TLR-mediated secretion of IL-12 by DCs triggers Stat4 phosphorylation and consecutive IFN-γ secretion in iNKT cells (175).

These Th-polarizing cytokines produced by NKT cells influence the outcome of naive T-cell differentiation (150). The interaction of different human iNKT cell subsets with DC can influence the polarization of T-cells toward different subsets. For example, when double-negative NKT cells interact with α-GalCer-loaded DCs, they produce IL-5 and IL-13 cytokines, typically produced by Th2 cells. In contrast, the interaction of CD4^+^ NKT cells with α-GalCer-DCs leads to the generation of IFN-γ, typically produced by Th1 cells (176). Additionally, cytokines delivered by iNKT cells will promote the polarization of Th cells since they either increase or suppress the adaptive immune response and cell polarization that promotes immunity or pathogenesis.

### 4.3 Can iNKT cells determine the quality of B cell responses?

It is generally accepted that, unlike classical Tfh cells, iNKTfh cell help cannot promote long-lived plasma cells and B-cell memory formation (24, 141, 144). However, two independent studies have demonstrated otherwise. Immunization with liposomes containing α-GalCer analogue PBS57 (which elicit both IFN-γ and IL-4 production) and coated with a polysaccharide derived from *Streptococcus pneumoniae*, promoted the production of IgM, IgG3, IgG1 and IgG2c, where this last one was induced after a boost (148). In this study, the absence of CD1d expression on DCs and B cells impaired the production of IgG1. Interestingly, there was no induction of iNKTfh cells (PD-1^+^ CXCR5^+^), but rather there was an induction of PD-1^+^ ICOS^+^ iNKT cells. This two-dose stimulation resulted in the induction of a long-term memory response (148). Since polysaccharide antigens usually don’t trigger Th cell activation, its suggested that this response was uniquely dependent of iNTK cells.

Similarly, immunization with liposomes containing α-GalCer and coated with ovalbumin antigen showed an increase in the avidity of OVA-specific antibodies, suggesting the generation of SHM and therefore affinity maturation. Additionally, in the absence of Tfh cells, these nanoparticles were capable of inducing the generation of memory iNKTfh cell and promoted recall immune response (149). Notably, the generation of memory iNKTfh cells required interaction solely with DCs, whereas B cells were crucial for germinal center formation and secondary antibody responses (149). This has also been reported with liposomes containing α-GalCer and a protease derived from MERS Coronavirus, where this formulation promoted strong antigen-specific humoral and cellular immune response inducing a memory response after a second immunization (177).

This highlights the importance of using particulate delivery of glycolipid antigen, and specifically liposomes coated with B cell antigen, either T-dependent or independent, to induce memory response. In these cases, coated antigens would probably enhance their recognition and uptake by B cells through BCR promoting concomitant activation of B and iNKT cells. These requirements would be necessary to induce stable and prolonged germinal center, where memory B cells are usually generated (120). Studies have shown that particulate antigens, like virus-like particles (VLPs) or liposomes, mimic natural pathogens by presenting repetitive epitopes that enhance BCR cross-linking, thus it has been seen that bacterial phage Qβ-derived virus-like particles (Qβ-VLPs) could induce Bcl-6 expression in pre-germinal center B cells independently of T cell help, and lead to isotype-switched and somatically mutated memory B cells (178). On the other hand, coengagement of BCR and TLR receptors on B cells, has been shown to induce CSR, SHM, germinal center-like differentiation, neutralizing antibodies and memory response (179). Therefore, the particle-based co-administration of glycolipids and B cell antigens may enhance the generation of memory iNKTfh cells, thereby promoting prolonged germinal center activity and memory formation. Additionally, this approach could facilitate the co-engagement of BCR and TLR receptors, which, in conjunction with the iNKT cell response, might further amplify the humoral immune response. However, this hypothesis requires further validation.

On the other hand, a previous study has shown that iNKT cell-derived BAFF and APRIL were critical for the maintenance of antibody titers after stimulation with NP-KLH/α-GalCer, therefore, promoting memory plasma cell survival (147, 180). Further research is needed to determine whether particulate antigen delivery enhances the production of BAFF and APRIL by iNKT or iNKTfh cells, or if α-GalCer analogues differentially regulate the secretion of these cytokines. Such investigations will provide deeper insights into the mechanisms underlying iNKT-mediated immune responses and aid in the design of more effective strategies for inducing protective immunity.

The importance of iNKTfh cells induction for a protective immune response has also been addressed in the context of T-independent antigens such as NP-Ficoll and *Clostridium difficile*-derived polysaccharide (181). Mice lacking Bcl-6 and therefore iNKTfh were unable to generate class-switched antibodies after immunization with α-GalCer and NP-Ficoll, and in addition, the absence of IL-21 also resulted in a reduced antibody response (182).

In line with this, Chang and colleagues showed differential induction of iNKTfh cells with α-GalCer analogues. The glycolipid OCH was unable to induce the generation of iNKTfh cells compared to α-GalCer, whereas α-C-GalCer promoted a higher expansion of this population compared to α-GalCer (24). While further validation with other α-GalCer analogues is necessary, these findings suggest that Th1-biased or iNKT1-activating glycolipid analogues are potent inducers of iNKTfh cells. As previously proposed, this could promote the stable and sustained formation of germinal centers, ultimately supporting the development of long-lived plasma cells and memory B cells. Of note, it has also been described that memory response can be induced in a GC-independent manner (183).

Interestingly, a recent study demonstrated that during the early phase (three days) following vaccination with pneumococcal surface protein A and α-GalCer, Gr-1^+^ CD11b^+^ monocytes and macrophages in the spleen’s red pulp promote iNKT cell activation, proliferation, and differentiation into iNKTfh cells (CXCR5^+^ PD-1^+^), which produce IL-4 and IL-21 (184). This process is mediated by IL-27 production by Gr-1^+^ cells, stimulating mitochondrial metabolism in iNKT cells required for their differentiation. Notably, IFN-γ secretion by iNKT cells enhances IL-27 production by Gr-1^+^ cells, as IFN-γ neutralization with an anti-IFN-γ antibody significantly reduced IL-27 levels (from ~9% to ~3%) and completely abolished iNKTfh cell formation. This vaccination strategy conferred protection against systemic S. pneumoniae infection, however further studies are require to clarify the dependency of pneumococcal surface protein A in the mechanism of iNKT cell differentiation, and also the role of iNKTfh cells in the immune response generated against this pathogen (184). Beyond highlighting the critical role of iNKT cells, cytokines, and innate immune cells in shaping an effective immune response, these findings reveal a novel mechanism of iNKTfh differentiation. This mechanism aligns with the previously mentioned association between Th1-biased α-GalCer analogues and iNKTfh formation, suggesting that Gr-1^+^ APCs and IL-27 production may play a key role in this process.

### 4.4 Can iNKT cells direct the Ig isotypes and subtypes produced by B cells?

Another question addressed in some reports is whether iNKT cell ligands, such as α-GalCer or its analogues, can modulate CSR through cytokine production, thereby influencing the antibody composition when used as adjuvants in responses to model or pathogen-derived antigens.

**Figure 3:**
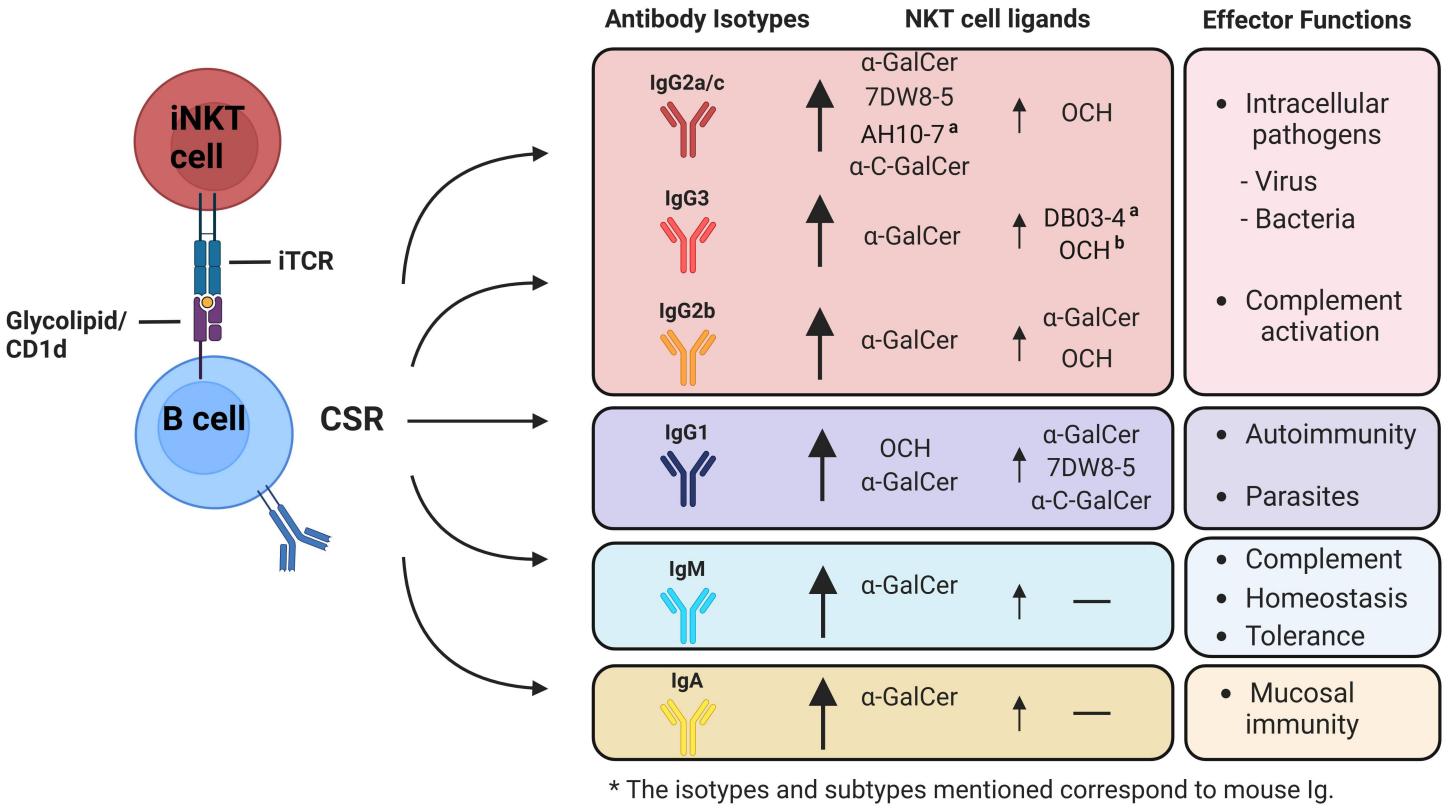
Possible role of α-GalCer or its analogues in the activation of iNKT cells and the modulation of B cell class-switch recombination (CSR) towards different antibody isotypes. Co-administration of protein or polysaccharide antigens together with glycolipid ligands ofi NKT cells, enhances the humoral immune response and influences the induction of class-switch recombination (CSR), resulting in different antigen-specific immunoglobulin isotypes based on the cytokine profiles secreted by iNKT cells subsets. Upon activation with α-GalCer, iNKT cells produce a combination of Th1-biased and Th2-biased cytokines, such as IFN-γ and IL-4, resulting in the so called Th0 cytokine profile. This profile generates a diverse array of antibody isotypes at varying levels. iNKT1-inducing or Th1-biased analogues of α-GalCer, like 7DW8-5 and α-C-GalCer, strongly stimulate the production (indicated by a big arrow) of IgG2a/c (depending on mouse strain), IgG2b, and IgG3. These analogues also induce IgG1 production to a lesser extent (indicated by a small arrow), which is associated with IL-4. In contrast, iNKT2-inducing or Th2-biased analogues of α-GalCer, such as OCH, primarily promote the production of IgG1 and to a lesser extent IgG2a/c, IgG2b, and IgG3. Isotypes associated with proinflammatory responses are well-documented for their role in pathogen elimination and control. In contrast, anti-inflammatory associated isotypes are recognized for their involvement in autoimmune disorders, and parasite control. Very few studies have investigated the impact of α-GalCer analogues on the induction of IgA antibodies. (a) Additional analogues that, even though they can induce polarized cytokine secretion, have not been studied for their influence on generating various antibody isotypes. (b) While not yet explored in the generation of IgG3, it is hypothesized that these analogues might lead to the mentioned effects. *The isotypes and subtypes mentioned correspond to mouse Ig.

As mentioned previously, it is known that IFN-γ drives the production of IgG1 and IgG3 subtypes in humans, whereas in mice it generates the production of IgG2a/c and IgG2b (168, 185–187). These subtypes have undergone thorough characterization due to their effector functions associated with opsonization, phagocytosis, and complement activation. Therefore, are essential in the context of bacterial and viral infections, making these IgG subtypes highly effective in combating infectious diseases (**Figure 3**) (188, 189).

On the contrary, IL-4 and IL-21 are involved in the production of IgG4 in humans, and IgG1 in mouse, whereas only IL-4 is implicated in the generation of IgE (168, 190, 191). As to IgG1, this isotype has been associated in the immune response against extracellular pathogens, such as helminths; whereas IgE is produced in response to allergens, therefore, mediating allergic reactions (**Figure 3**) (192).

In this regard, C57BL/6 mice immunized with α-GalCer together with tetanus toxoid (TT), diphtheria toxoid (DT), or influenza H3N2 antigen, enhanced antibody production in an iNKT-dependent manner. As to the antibody isotypes produced, the main IgG subtypes produced were IgG1 and IgG2c, and at lower levels IgG2b and IgG3 (31). This has been reported in other studies (31, 34, 149). To address the implications of IL-4 and IFN-γ in antigen-specific IgG subtype production, IL-4^-/-^ or IFN-γR^-/-^ mice were immunized showing that in the absence of IL-4, the production of IgG1 and IgG2a wasn´t affected, whereas in the of IFN-γ receptor, only IgG2a production was profoundly affected (31). As to other isotypes, α-GalCer has also been shown to induce IgA, which is proposed to me induced by TGF-β and retinoic acid (193–195).

The analogue 7DW8-5 has exhibited a more prominent adjuvant effect than α-GalCer, resulting in a robust humoral response when administered alongside HIV and Malaria vaccines. However, the precise composition of the induced antibodies was not specified (64). The intranasal administration of 7DW8-5 before SARS-CoV-2 infection has demonstrated significant efficacy in preventing infection by this virus, and this effect was dependent on CD1d and IFN-γ. Moreover, this approach has also effectively countered infections caused by respiratory syncytial and influenza viruses (67). Similar studies have shown that this analogue was able to induce both IgG2a and IgG1 in BALB/c mice when being used as an adjuvant in the co-administration of a commercial influenza HA vaccine, reflecting the induction of both Th1-like and Th2-like immune response, however, the genetic background of BALB/c mice associated to a Th2 response could highly influence the production of IgG1 (196, 197).

α-C-GalCer was also evaluated in this regard. Its adjuvant effects were assessed when co-administered with a live attenuated influenza virus vaccine in BALB/c mice. This showed a pattern similar to that of 7DW8-5, inducing Th1-like and Th2-like associated isotypes, with IgG2a levels being more pronounced than IgG1, resulting in reduced morbidity and mortality after a challenge with the virus (61). Despite these results, further assays are required using different mice strains to evaluate the full potential of these analogues in CSR.

On the contrary, the use of Th2-biased analogues will induce the production of IL-4, which is involved in the generation of IgG4 and IgE in humans, and IgG1 and IgE in mouse (190, 191). As to IgG1, this isotype has been associated in the immune response against extracellular pathogens, whereas IgE mediates immune response against parasites and also is implicated in the induction of allergy (**Figure 3**) (192).

The analogue OCH has been reported to induce higher levels of IL-4 and lower levels of IFN-γ compared to α-GalCer, and it is being evaluated for treating experimental autoimmune encephalomyelitis (EAE) in mice. The administration of α-GalCer induces the production of IFN-γ, promoting the generation of both IgG1 and IgG2a at the same levels. On the other hand, the OCH glycolipid shifts the response towards a Th2-like profile, enhancing the production of IgG1 over IgG2a, in which the overall response results in the suppression of EAE (198).

The same tendency was observed in prevention of insulitis and diabetes in NOD mice, where the administration of OCH influenced the humoral response generated against autoantigens (anti-GAD antibodies) as part of the autoimmune condition, promoting a significant increase in the ratio between IgG1 and IgG2a compared to α-GalCer. Overall, this study showed that OCH could prevent the development of diabetes and insulitis in this mouse model (199).

Despite these reports, many α-GalCer analogues remain unexplored in their capacity to induce or regulate CSR toward other antibody isotypes, particularly IgE and IgA. Addressing these gaps would enhance our understanding of iNKT-B cell interactions in various pathologies and contribute to vaccine development and therapeutic approaches (**Figure 3**). Furthermore, the impact of α-GalCer analogues on germinal center formation and the generation of memory humoral responses remains an open question. Based on the information presented, we propose that targeting specific iNKT cell subsets using tailored α-GalCer analogues could be a novel and effective strategy to modulate humoral responses. This approach could facilitate the induction of specific antibody isotypes, as well as promote germinal center formation and memory responses, thereby highlighting the immunotherapeutic potential of iNKT cells.

Additionally, although efforts have been made to develop vaccines containing α-GalCer analogues targeting viruses such as SARS-CoV-2 and Influenza, as well as bacterial pathogens like S. pneumoniae and C. difficile, with varied results, further research is needed on germinal center formation, antibody isotype switching, and the specific iNKT cell subsets involved in these responses (61, 67, 146, 148, 181). A deeper understanding of these mechanisms is crucial to improving immune responses against these pathogens and advancing the development of more effective vaccines.

### 4.5 iNKT cells shape antibody composition, immune regulation, and disease pathogenesis in humans

Human B cells are heterogeneous. The main subsets identified are B1 (CD5^+^), mature (CD22^+^), naïve (CD27^-^IgD^+^), plasma cells (CD38^hi^) and memory (CD27^+^), and among memory, these can be classified as unswitched memory (CD27^+^IgD^+^), switched memory (CD27^+^IgD^-^) and CD27^-^ memory B cells (CD27^-^IgD^-^) (200). CD1d expression is uniform among different subsets, ranging from 60 to 80% CD1d-expressing B cells (201).

Human iNKT cells are located mainly within the thymus, liver, bone marrow, spleen, and peripheral blood (40). Similar to B cells, human CD3^+^Vα24^+^Vβ11^+^ iNKT cells can be divided into different subsets based on the expression of CD4 and CD8 coreceptors. CD4^+^CD8^-^, CD4^-^CD8^+^, and CD4^-^CD8^-^ (double negative) subsets (202, 203). Interestingly, CD8^+^ subset is found in humans and rats, but not in mice (202, 204, 205). The effector phenotype of CD4^+^ iNKT cells has been associated with iNKT2 cells, since they produce mainly Th2-associated cytokines such IL-4, whereas CD8^+^ and double negative iNKT cells are associated with iNKT1 cells, given that they exhibit predominantly a Th1-associated phenotype with IFN-γ production and cytotoxic activity (202, 203, 206). Of note, iNKT cell frequency is very low, between 0.003-0.71% CD3^+^/Vα24^+^/Vβ11^+^ cells and 0.019-0.776% CD3^+^/6B11-stained cells (monoclonal antibody that recognize an epitope of the CDR3 formed by the germ-line configuration of the Vα24 and Jα18 of the TCRα locus) of peripheral blood T cells of healthy Caucasian children from 7 months to 18 years of age (207). In healthy adult individuals, the frequency of circulating iNKT cells ranges between 0.01-0.92%, based on the staining with 6B11 mAb, with no differences between male and female subjects (208).

One of the first reports addressing iNKT cell impact on human B cell functions were made by Galli et al. (209). In these assays, culturing sorted iNKT cells with autologous CD20^+^, CD20^+^CD27^+^ (memory), or CD20^+^CD27^−^ (naïve) B cells for five days led to the expansion of these subsets in a CD1d-dependent manner, with α-GalCer further enhancing proliferation. IgM production was strongly dependent on the presence of both α-GalCer and CD1d, although polyclonal stimulation of iNKT cells with anti-CD3 antibodies induced a modest increase. Regarding IgG production, only the IgG1 subtype was evaluated. α-GalCer significantly increased IgG1 levels in a CD1d-dependent manner; however, CD1d blockade did not completely inhibit IgG1 production, mirroring the partial effect observed with anti-CD3 stimulation. Additionally, comparisons between CD4^+^ and double-negative iNKT cells in B cell activation revealed that both subsets expressed basal levels of CD40-L. While both subsets promoted B cell expansion and CSR, CD4^+^ iNKT cells demonstrated superior IgM and IgG1 production (209). These results correlate with the iNKT2-associated phenotype of CD4^+^ iNKT cells. Of note, IgE production was not detect in this context, although it has been described in other studies using CD4^+^ iNKT cells (201, 210). These findings suggest that optimal human B cell activation requires direct interaction with iNKT cells, complemented by costimulatory signals and cytokine production, to effectively drive activation and induce CSR.

Another study by Zeng, et al. has also described differential outcomes on the interaction of iNKT cell subsets and B cells (201). For instance, human CD4^+^ iNKT cells cocultured with B cells were capable of inducing the production of IgM, IgG and IgA, in the absence of α-GalCer, whereas CD8^+^ subset promoted the production of IgM and IgG, and double negative subset only promoted IgM production. Additionally, CD4^+^ subset induced the expansion of CD1d^+^CD5^+^ b cells and only modest increase of CD24^HIGH^CD38^HIGH^ B cells, and also promoted the upregulation of CD40 and CD86 in the presence of α-GalCer. Interestingly, double negative iNKT cells displayed a significant increase of CD107a presumably to kill autoreactive B cells. Of note, fewer than 2% of three distinct iNKT populations were able to produce IL-21, a hallmark of the iNKTfh phenotype (201). Although iNKT cells enhanced the expression of costimulatory molecules on B cells, the findings suggest that the response may occur in an extrafollicular manner, given the absence of iNKTfh cells.

Further characterization of B and iNKT cell dynamics in human contexts is challenging due to limited sample availability, the low frequency of these cells in peripheral blood, and the constraints of *in vitro* functional assays, which fail to fully replicate the complexity of cellular interactions in humans (211, 212). Recent efforts to address these challenges have focused on developing partially humanized mouse models that more accurately replicate human iNKT cell frequency, distribution, and function, enhancing the translation of findings to human contexts. One of the earliest models developed was the human CD1d (hCD1d) knock-in mouse, where the mouse CD1d gene was replaced with the human counterpart. In this model, hCD1d is expressed in a native tissue distribution pattern, supporting the development of iNKT cells that closely mirror human iNKT cells in frequency, phenotype, and reduced CD4 expression. The responding iNKT cells predominantly express Vβ8, homologous to the human Vβ11 rearrangement (213). Additionally, iNKT cells in this mouse model demonstrated strong antitumor activity (68, 213). Further humanization led to the replacement of mouse invariant TCRα-chain with the orthologous human Vα24Jα18 invariant TCRα-chain into hCD1d mice (214). This humanized mouse model developed a subset of CD8^+^ iNKT cells, akin to those found in humans, originating in the thymus. This subset exhibited a Th1-biased cytokine response and demonstrated cytotoxic activity against tumor cells, highlighting the model’s ability to replicate the phenotypic and functional characteristics of human iNKT cells (214).

While these advances mark significant progress in developing robust humanized mouse models, Porcelli’s group has recently introduced a novel model defined as VαKI (215). This mouse model features a deletion of the Jα18 locus (*Traj18*) to specifically eliminate the expression of endogenous mouse iNKT cell invariant TCRα chains. As a result, it develops functional iNKT cells with frequencies, phenotypes, and functions closely resembling those of humans, while maintaining a normal immune system, including conventional T cells. Notably, its activation pattern closely mirrors that of human iNKT cells, with the analogue 7DW8-5 inducing stronger activation compared to AH10-3 and other analogues, as evidenced by increased IFN-γ production and antitumoral activity (215).

The use of these mouse models offers a powerful tool to study iNKT cell dynamics, enabling the evaluation of humoral response processes such as CSR, germinal center formation, and memory responses. Additionally, it provides a platform to assess the potential effects of α-GalCer analogues on these processes, which could be pivotal for optimizing translational therapies. This was already addressed by Saavedra-Avila et al., who utilized computational analysis to identify an α-GalCer analogue with higher stimulatory activity in VαKI mice, which had been overlooked in earlier studies in WT mice (71).

The use of α-GalCer in clinical trials has been proven to induce therapeutic effects. α-GalCer-pulsed DCs when administered in twelve patients with metastatic malignancy lead to activation of both innate and adaptive immunity, resulting in the modulation of NK, T and B cells, and increased serum levels of IFN-γ (216). A Phase I/II trial involving forty patients with chronic hepatitis C demonstrated that α-GalCer, administered at doses ranging from 0.1 to 10 μg/kg, was well tolerated without causing any side effects. However, these doses were ineffective in eliminating HCV-RNA levels (217). As mentioned in the previous sections, OCH was recently used in the first-in-human clinical trial for the treatment of multiple sclerosis, showing to be safe and with anti-inflammatory effects (55).

The role of iNKT cells in pathological contexts has also been addressed extensively (218–224). Metabolic disorders associated with obesity lead to the accumulation of T-bet^+^ B cells in human adipose tissue, a process supported by IFN-γ-producing iNKT cells. These T-bet^+^ B cells contribute to inflammation and exacerbate metabolic dysfunction by producing IgG2c antibodies and the chemokine CXCL10 (225). It remains unclear whether T-bet^+^ B cells receive assistance from iNKTfh or Tfh cells via cytokine production and costimulatory signals, or if the class-switched IgG2c-producing T-bet^+^ B cells arise from germinal center formation or extrafollicular interactions.

Recent reviews highlight how viral infections often regulate CD1d expression, thereby influencing iNKT cell-mediated immune responses (224, 226). Subjects with iNKT cell deficiencies or reduced CD1d expression have intensified symptoms after viral infections (227–229). During the recent COVID-19 pandemics, data showed that patients with SARS-CoV-2 infection had reduced iNKT cell in peripheral blood, which expressed higher levels of the exhaustive marker Tim-3 (230, 231). Similarly, incubation of human iNKT cells with HSV-1 infected human keratocytes impaired iNKT cell activation both through cytokine- and TCR-dependent activation (232). In the context of dengue virus, NKT cell deficiency skews the immune response, leading to elevated levels of Th2-associated IgG1 over Th1-associated IgG2a. This imbalance fails to provide protection against homologous DENV rechallenge and promotes antibody-dependent enhancement of disease during secondary heterologous infections. Similarly, in humans, Th2-dominated immunity, characterized by a higher IgG4/IgG3 ratio, has been linked to increased disease severity during secondary dengue infections (233).

In certain pathologies caused by human herpesvirus 8 (HHV-8) infection, where iNKT cell frequency is reduced, there is also a lower number of circulating MZ B cells and memory B cells (CD27^+^IgD^+/-^) (234). Conversely, apoE-deficient (apoE^-/-^) mice, with inefficient lipid capture and CD1d presentation by DCs activation, show increased MZ B cells related to a decreased apoptotic cell death (235). Thus, iNKT cells could be relevant in maintaining a correct balance in B-cell subsets.

On the other hand, genetic pathologies related to antibody production such as Common variable immunodeficiency (CVID) have also been linked to iNKT cell function. CVID is the commonest symptomatic primary antibody deficiency, in which most of the patients with this pathology have a reduced number of memory B cells and failure of antibody production, characterized by reduced levels of serum IgG, IgA, and in some cases of IgM, making them highly susceptible to infections (236, 237). Patients diagnosed with CVID have reduced number of iNKT cells compared to healthy individuals, in which their phenotype was predominantly CD4^+^, with a higher and lower number of IFN-γ and IL-17-producing cells, respectively, compared to control after PBMC stimulation with α-GalCer (238), although other reports show otherwise (239). Additional studies have examined whether the reduced frequency of iNKT cells is more pronounced in patients who also exhibit decreased frequencies of isotype-switched memory B cells. However, findings have been conflicting, with reports presenting opposing results (240, 241).

Further studies aiming to characterize iNKT cells in patients with CVID showed reduced number of CD4^+^, double negative, and CCR5^+^/CXCR3^+^ iNKT cells in blood, together with higher frequency of CD40-L^+^ iNKT cells and iNKTfh cells, compared to healthy individuals (241). In addition, reduced expression of SAP was observed in iNKT, NK, and T cells of CVID patients compared to healthy individuals, which could be associated with the retention of high number of iNKTfh cells in the peripheral blood of these patients (241). Moreover, additional experiments are necessary to evaluate whether iNKT cells can interact and induce B cell activation in these patients. Additional perspectives on this topic have been addressed elsewhere (242).

Therefore, iNKT cells play a crucial role in modulating B-cell functions and antibody responses, influencing immune regulation in health and disease. Their interactions with B cells, driven by cytokines, costimulatory signals, and direct contact, are pivotal in shaping CSR and antibody composition. Pathologies like obesity, viral infections, and autoimmune conditions reveal how iNKT cell dysfunction can lead to imbalanced humoral responses, exacerbating disease severity. Advancements in humanized mouse models and computational tools provide valuable platforms to study these mechanisms and explore the therapeutic potential of α-GalCer analogues. Understanding the impact of antibody composition on disease progression remains essential for improving immunotherapies, vaccines, and translational medicine.

## 5 Concluding remarks

Unlike classical Tfh cells, iNKT cells offer unique mechanisms to optimize humoral immunity through their innate-like rapid activation by glycolipid antigens presented on CD1d molecules. Their ability to engage in direct synaptic interactions with B cells and other APCs such as DCs, and macrophages, coupled with diverse cytokine production and costimulatory molecule expression, highlights their versatility as modulators of immune responses.

During the early stages of an immune response, distinct APCs may engage iNKT cells using specific costimulatory molecules. Each type of APC influences the activation of different iNKT cell subsets, as evidenced by the observed cytokine profiles. For instance, MZ B cells predominantly mediate the production of IL-4 and IL-13, likely driven by iNKT2 activation through ICOS-ICOSL and PD-1-PD-L1/PD-L2 signaling. Conversely, DCs promote the production of both IFN-γ and IL-4, indicative of iNKT1 and potentially iNKT2 activation, via pathways such as CD28-CD80/CD86, CD27-CD70, and NKG2D-Rae-1. These findings highlight the potential for multiple APCs to synergistically enhance iNKT cell responses, collectively shaping their immunological outcomes in the early response. Despite this proposed model, the precise relationships between APCs, cytokine outputs, and the full spectrum of iNKT subset activation remain incompletely characterized. A comprehensive analysis of the expression of signature transcription factors and cytokines specific to each iNKT subset is essential to fully understand these interactions. iNKT cells likely interact with MZ B cells through both cell contact-dependent and contact-independent mechanisms, thereby influencing the characteristics of extrafollicular antibody production, promoting CSR, and potentially inducing the formation of transient germinal center B cells. In this regard, iNKT cells rely on B cells for their differentiation into iNKTfh cells, a process that enables their migration to follicles, where this differentiation may be driven by interactions with MZ B cells considering that they differentiate into FO B cells and migrate to follicles. Within the follicles, iNKTfh cells interact with FO B cells, influencing their activation and the subsequent germinal center responses. Interestingly, various α-GalCer analogues have been shown to elicit distinct effects on iNKT cell differentiation into iNKTfh cells, specially Th1-biased analogues such as α-C-GalCer, which has been proved to induce a higher frequency of this phenotype compared to α-GalCer, whereas OCH didn´t have an impact on this cells, therefore the use of Th1-biased analogues may impact the quality and outcome of the humoral immune response.

On the other hand, recent findings emphasize that glycolipid-based particulate delivery systems, particularly liposomes, enhance germinal center dynamics and memory responses by promoting iNKT cell activation, and possibly inducing iNKTfh generation. This approach has been shown to promote CSR and SHM, and although it is still very controversial, it also promoted robust and long-lasting antibody responses inducing memory B cells.

Furthermore, the activation of different iNKT cell subsets using various α-GalCer analogues may influence the specific antibody isotypes or subtypes, particularly within the IgG class, produced in response to clinically relevant antigens. Since each isotype plays a distinct role in immune responses, the use of these analogues could offer a promising approach for vaccine development.

Despite significant progress, knowledge gaps persist regarding the role of antibody composition and class switching in disease pathology, particularly in contexts such as obesity, viral infections, and autoimmune conditions. Although advanced humanized mouse models and computational tools help to replicate and analyze human-specific iNKT cell activation, further strategies are required to integrate this info into B cell activation and humoral responses.

A deeper understanding of iNKT cell biology and its influence on B-cell dynamics could enhance immune responses across diverse clinical contexts. These advancements promise to optimize vaccine strategies, advance targeted immunotherapies, and address unmet challenges in translational medicine, ultimately improving outcomes across a broad spectrum of diseases.
